# Dendritic Cells, the Double Agent in the War Against HIV-1

**DOI:** 10.3389/fimmu.2019.02485

**Published:** 2019-10-23

**Authors:** Alba Martín-Moreno, Mª Angeles Muñoz-Fernández

**Affiliations:** ^1^Sección de Inmunología, Laboratorio InmunoBiología Molecular, Hospital General Universitario Gregorio Marañón (HGUGM), Madrid, Spain; ^2^Instituto Investigación Sanitaria Gregorio Marañón (IiSGM), Madrid, Spain; ^3^Spanish HIV-HGM BioBank, Madrid, Spain; ^4^Networking Research Center on Bioengineering, Biomaterials and Nanomedicine (CIBER BBN), Madrid, Spain

**Keywords:** dendritic cells, HIV-1, endocytosis, trans-infection, cis-infection, immune response

## Abstract

Human Immunodeficiency Virus (HIV) infects cells from the immune system and has thus developed tools to circumvent the host immunity and use it in its advance. Dendritic cells (DCs) are the first immune cells to encounter the HIV, and being the main antigen (Ag) presenting cells, they link the innate and the adaptive immune responses. While DCs work to promote an efficient immune response and halt the infection, HIV-1 has ways to take advantage of their role and uses DCs to gain faster and more efficient access to CD4^+^ T cells. Due to their ability to activate a specific immune response, DCs are promising candidates to achieve the functional cure of HIV-1 infection, but knowing the molecular partakers that determine the relationship between virus and cell is the key for the rational and successful design of a DC-based therapy. In this review, we summarize the current state of knowledge on how both DC subsets (myeloid and plasmacytoid DCs) act in presence of HIV-1, and focus on different pathways that the virus can take after binding to DC. First, we explore the consequences of HIV-1 recognition by each receptor on DCs, including CD4 and DC-SIGN. Second, we look at cellular mechanisms that prevent productive infection and weapons that turn cellular defense into a Trojan horse that hides the virus all the way to T cell. Finally, we discuss the possible outcomes of DC-T cell contact.

## Introduction

Human Immunodeficiency Virus (HIV) infects cells from the immune system, its main target being CD4^+^ T cells. For this purpose, the virus has developed tools to circumvent the host immunity and even use it in its advance. The disease progression can differ dramatically depending on the first interaction between the virus and the immune cells, and the early host response, which can produce neutralizing antibodies and cytotoxic responses. HIV-1 hijacks the host's immune mechanisms and uses them to spread faster, resulting in a two-edged fight by the immune system, stuck between not responding or responding and facilitating the viral spread.

Nowadays, and only for the lucky patients who have access to treatment, HIV-1 infection is a chronic disease instead of a death sentence. However, less than 60% of the 37 million people infected with HIV-1 are on antiretroviral therapy according to UNAIDS data. Adherence to uninterrupted treatment is vital, as viral load and lymphocyte death rapidly increase upon treatment interruption. Economic issues and a wide range of social and psychological side effects result in scarcity of treatment available or lack of adherence to treatment. For these reasons, finding a more accessible and long-lasting solution against HIV-1 is one of the biggest current challenges of the biomedical community.

Viral latency, high mutability and variability highly hinder the finding of a cure or an efficient prophylactic vaccine (1–3), making the possibility of either option controversial among researchers (4, 5). Therefore, finding a functional cure appears as a very appealing option. Functional cure means that HIV-1 is still present in the host organism and remains latent in the genome of many cells, but the host immune system keeps the infection under control in the absence of treatment, which requires a specific and efficient immune response.

The idea of modulating the immune system as a way to fight disease was first formulated in the late 19th century by William B. Coley, and it is now known as immunotherapy or biological therapy. It is often successfully used in cancer therapies, which use immune modulators to halt the growth of cancer cells, and to enhance the cytotoxic immunity and cancer recognition to fight the tumor. The protocols for *ex-vivo* (in a laboratory) or *in-vivo* modulation of the patient's immune cells are rapidly increasing in the era of personalized medicine.

Due to their role as antigen presenting cells (APCs), dendritic cells (DCs) are promising candidates to achieve the functional cure of HIV-1 infection. DCs are innate immune cells that patrol tissues, recognize Ag, participate in early immune response, and, upon Ag uptake and processing, present Ag and activate T cells, serving as a link between general innate immunity and specific adaptive immune cells. DCs are localized in all tissues in the body, and undergo maturation and migrate to the lymph nodes upon encountering an Ag (6, 7). Once in the lymph nodes, they connect with naïve T cells through what is known as immune synapse, which serves to both present Ag and activate the lymphocyte. If this process is successful, it triggers a specific immune response (8). However, HIV-1 also exploits DCs as a means of transportation from the site of infection to the lymph nodes, where the high density of CD4^+^ T cells and direct cell-to-cell contact through immune synapses ease the spread of the virus and fast infection of a high number of cells.

In order to successfully design a DC-based immunotherapy, it is essential to understand all the diverse interactions between DCs and HIV-1, and the factors that determine the outcomes of those interactions. In this review, we summarize the current state of knowledge on DCs and their role and behavior during HIV-1 infection.

## Dendritic Cells

Dendritic cells represent 0.5–2% of peripheral blood mononuclear cells (PBMCs) (9). DCs are less susceptible to HIV-1 infection than CD4^+^ T cells, as only around 1% of DCs are infected (10), and the HIV-1 infection is less productive than in CD4^+^ T cells. Nonetheless, DCs are of utmost importance for the immune response to HIV-1 as they are among the first cells to encounter the virus after the infection through the mucosa and play a pivotal role in the establishment of HIV-1 infection, and progression of the disease (11).

Immature DCs (iDCs) are located in the mucosa and peripheral tissues, where they capture and process antigens. The encounter of an iDC with the stimulus of an Ag causes the maturation and the subsequent migration of the now mature DCs (mDCs) to the secondary lymphoid tissues, where they present the Ag to lymphocytes and prime naïve T cells (12, 13).

As key immune cells, DCs secrete a diverse group of interleukins, aimed to orchestrate the immune response. Most of these cytokines, including IL-2, IL-7, IL-12, IL-15, IL-18, IL-23, and IL-27, induce or enhance maturation, activation and proliferation of Th1 cells, and cytotoxic responses. DCs also secrete the immunosuppressive IL-10 (14).

Classically, DCs were described as HLA-DR^+^ lineage^−^ cells, due to the high expression of major histocompatibility complex (MHC) class II (HLA-DR) and the lack of typical lineage markers, such as CD3 (T cells), CD19/20 (B cells) and CD56 (Natural Killer (NK) cells). However, more recently different subtypes of DCs were identified, and a number of DCs lineage markers were recognized (15). Nowadays, there is some consensus on this topic, and, as it has been recently reviewed by Rhodes et al. (16) and Collin and Bigley (17), DCs are divided in three well-differentiated subsets with specific functions and characteristic markers. This classification recognizes plasmacytoid DCs (pDCs) and two types of “classical” or “conventional” DCs (cDCs), previously known as myeloid DCs (15, 18, 19), known as cDC1 and cDC2 (Table 1).

**Table 1:** Comparison between plasmacytoid and conventional DCs.

	**Plasmacytoid DCs**	**Myeliod/conventional DCs**	
		**cDC1**	**cDC2**
Origin	Plasmacytoid cells	Myeloid precursor cells or Axl^+^ cells	
Markers	CD303 (CLEC4C) CD304 (neuropilin) CD123 (IL-3R) BDCA-2 BDCA-4 LILRA4	CD13, CD33 CD11b,c (low) SIRPa (low) CD1a,b,c, and d CD209 (DC-SIGN) BDCA-3 (Clec)9A CADM1 FactorXIIIA	CD13, CD33 CD11b,c SIRPa CD1c, CD2 FceR1 Clec4A,10A,12A,13B VEGFA CD32a CD14
TLR expression	TLR7, TLR9	TLR3, TLR9, TLR10	TLR2, TLR4, TLR5, TLR6, TLR8, TLR9
Chemokine receptors	CCR1, CCR2, CCR5, CCR9 CCR7 (mature cells) CXCR1, CXCR3	CCR1, CCR2, CCR4, CCR5, CCR6, CCR9 CCR7 (mature cells) CX3CR1	
Cytokine production	IFN-α, IFN-β IL-6, IL-8, IL-12, IL-18 TNF-α CCL3, 4, 5 CXCL10,11 CXCL11	IFN (type I) IL-12	IL-1, IL-6, IL-8, IL-10, IL-12, IL-15, IL-18, IL-23 TNF-α CCL3, 4 CXCL8
Main function	IFN production (activation of antiviral immune response)	Antigen presentation via MHC class I (priming and activation of CD8^+^ T cells)	Antigen presentation (lymphocyte priming and activation)
Anti-HIV-1 functions	Inhibition of viral replication (type I IFN)	Generation of specific adaptive immune response (mainly cytotoxic)	Generation of specific adaptive immune response (humoral and cytotoxic)
Pro-HIV-1 functions	T cell recruitment to infection site T cell activation (increasing susceptibility to HIV-1 infection) Long-term immune suppression (IDO production)	HIV-1 transport to lymph nodes Cell-to-cell transfer to T cells	

cDCs express the myeloid antigen CD1a, b, c, and d, together with CD14, CD209 (Dendritic Cell-Specific Intercellular adhesion molecule-3-Grabbing Non-integrin (DC-SIGN)), and Factor XIIIA, at expression levels similar to those of monocytes. A small subset of cDCs, the cross-presenting DCs, also expresses CD141, CLEC9A, and XCR1. However, pDCs are characterized by the expression of CD303 (CLEC4C), CD304 (neuropilin), and CD123 (IL-3 receptor) (20).

Both pDCs and cDCs display characteristic surface markers on their membrane whose expression levels correlate with their maturation/activation state: CD83 is a maturation marker; CD80 and CD86 are activation markers involved in Ag presentation and activation of T cells; and CCR7 is a chemokine receptor up-regulated in mDCs implicated in migration to secondary lymphoid organs (21). Furthermore, all DCs express the receptor that participates in binding and internalization of HIV-1, CD4, as well as the co-receptors CCR5 and CXCR4 (22).

### Plasmacytoid Dendritic Cells

pDCs present a lack of lineage markers, CD19-, CD3-, CD11c-, CD14-, but express MHC-II (HLA-DR^+^). pDCs can be recognized among DCs because they express selective markers including BDCA-4, BDCA-2, LILRA4, and CD123 (20).

Immature pDCs express the chemokine receptors CCR1, CCR2, CCR5, CCR6, and CXCR1. Thus, pDCs are recruited to areas of infection by chemokines such as MIP-3 alpha/CCL20 (23). After virus uptake via endocytosis, pDCs recognize RNA viruses (like HIV-1) via TLR7, activating a signaling cascade that results in pDCs maturation, IFN-α, IFN-β, IL-6, and TNF-α production, and changes in expression of membrane proteins, including an increase in expression of chemokine receptor CCR7 (24) and CD40, CD80, and CD86 co-stimulatory molecules. CCR7 has a key role in driving the maturing DCs to the lymph nodes. Expression of MHC-II and the co-stimulatory molecules allows pDCs to present Ags to CD4^+^ T cells, although not as efficiently as cDCs (25, 26). However, the most characteristic function of pDCs is type I IFN production, which stimulates a strong anti-viral response, but may also contribute to the chronicity of HIV-1 infection (27). Other soluble factors released by pDCs upon activation are CCL3, CCL4, CCL5, IL-8, CXCL10, and CXCL11 chemokines (28, 29). The multiple functions attributed to pDCs including IFN production, NK cell activation via IL-12 and IL- 18 secretion and Ag presentation have been reviewed by Swiecki and Colonna (30).

pDCs are among the first cells to encounter HIV-1 after infection (27). pDCs detect HIV-1 through TLR 7, which leads to activation, but can also be infected by HIV-1, due to the fact that they express CD4, CXCR4, and CCR5 (11). The role of pDCs in HIV-1 infection and disease development is both beneficial and detrimental because HIV-1 manipulates the anti-viral mechanisms of the cell to its own benefit. The functions of pDCs in the HIV-1 infection can be simplified in four steps (27): first, pDCs produce high levels of type I IFN, inhibiting viral replication and inducing bystander T cell activation which means to be a defensive response but also leaves the lymphocytes susceptible to HIV-1 infection. Second, pDCs release chemokines such as CCL5 that recruit CCR5^+^ CD4^+^ T cells to the site of infection and thus facilitate the spread. However, it is also noteworthy that CCL5, similarly to Maraviroc and other CCR5 ligands, competes with HIV-1 protein gp120 for binding to CCR5 co-receptor, thus having a inhibitory effect on HIV-1 entry and infection (31). Third, during chronic HIV-1 infection, the production of IFN persists, causing apoptosis of T cells and contributing to decrease in CD4^+^ cell count. And fourth, pDCs produce the enzyme indoleamine-pyrrole 2,3-dioxygenase (IDO) that skews Treg/Th17 homeostasis toward the increase of immunosuppressive Treg cells (32).

Although the number of pDCs in peripheral blood drops during HIV-1 infection, in parallel with the drop of CD4^+^ T cells, the remaining pDCs maintain their phenotype and are, in fact, hyper activated. The decrease of pDC number in blood is believed to be caused partially because of high cell death, but also because they migrate to the lymph nodes and accumulate there, where they keep a high activation state that eventually leads to their own apoptosis (33, 34).

It has been shown that DCs from blood from HIV-1-infected individuals not only present elevated expression levels of TLR and their downstream components, but also they are hyper-responsive to TLR7 agonists and produce high levels of cytokines after stimulation (24). In the same study, they showed that pDCs from uninfected individuals up-regulate CCR7, CD40, and CD86 in response to TLR7/8 agonist R848 and aldrithiol-2 (AT-2)-inactivated HIV-1, and pDCs from HIV-1-infected individuals retain their capacity to up-regulate these receptors (24). Moreover, it has been shown that when comparing HIV-1-infected individuals and healthy controls, there is no difference in the expression of CCR7, CD83, CD80, and CD86 after TLR7 stimulation, and likewise, there is no significant difference in IFN-α production per cell (35).

Due to their role in IFN production, a dysregulation in pDC function may result in uncontrolled viral infections (36). For most viruses, a lower IFN production would result in a lower antiviral response and a stronger infection, but in the opposite case, when the IFN release is maintained in time, it would result in T cell apoptosis, and a decrease in the host defenses. More strongly, in the case of HIV-1 infection, IFN release is also responsible for T cell activation, rendering them susceptible to HIV-1 infection. Not only the IFN production, but also the multiple roles of pDCs make a pivotal difference in the fate of HIV-1 infection and viral spread or immune response.

### Conventional Dendritic Cells

Unlike pDCs, which mainly respond to pathogens by secreting large amounts of IFN-α, cDCs are specialized APCs that serve as an important link between the innate immune system and the adaptive immune response. Upon Ag recognition, cDCs produce different inflammatory cytokines, including IL-12, IL-15, IL-23, IL-6, TNF, and IL-1b (14). Similarly to pDCs, cDCs express the chemokine receptors CCR1, CCR2, CCR5, CCR7, and CCR9, and they also express CCR4, CCR6, and CX3CR1 (37). cDCs are rarely infected by HIV-1, but they capture and internalize virus. As we will explain in the next sections, internalized virus can be processed and presented in the cell surface for T cell priming, or can on the contrary be transferred to CD4^+^ T cells, promoting this way the HIV-1 infection and spread. Similar to pDCs, cDCs play a dual role, simultaneously restraining and potentiating HIV-1 infection (38).

During HIV-1 infection, cDCs are mostly depleted from the blood, partially because of apoptosis and partially because they migrate and accumulate in lymph nodes (39, 40). Moreover, the differentiation of cDCs from monocytes, which usually happens upon inflammation, may be impeded due to aberrant IFN-α production (41). The functionality of the remaining circulating cDCs is controversial. Some studies report that maturation of cDCs is impaired (42–44), whereas other studies claim that cDCs are not functionally defective, respond to TLR7 stimulation by up-regulating CCR7, CD40, CD80, CD83, and CD86 (21, 24), and may even be hyper-responsive to TLR stimulation (10). Binding and internalization of HIV-1 is mediated by a variety of DCs-expressed surface receptors. While activation of pDCs by HIV-1 is mainly thought to involve intracellular members of the TLR family, it has been shown that HIV-1 does not induce maturation of cDCs by activating TLRs (45, 46). HIV-1 also prevents the activating effect of cDCs on other cell types. In normal conditions, cDCs mediate NK cell activation and fuel its lytic activity, but HIV-1 disrupts cDC-NK cell cross talk, weakening this innate line of defense (47).

The few cDCs that are infected by HIV-1 sustain a substantial viral burden and have a relatively long half-life, which suggests that they may have a role in latency. HIV-1 can signal through TLR-8, which initiates transcription from integrated provirus via MAPK pathway and nuclear factor kB (NF-kB) (48).

It has been observed that early HIV-1 infection induces a strong and simultaneous increase of LILRB2 and MHC-I expression on the surface of blood cDCs. Analysis of factors involved indicates that HIV-1 replication, TLR7/8 triggering, and treatment by IL-10 or type I IFNs increase LILRB2 expression (49). *In vitro* experiments suggest that strong LILRB2-HLA binding negatively affects Ag-presenting properties of DCs (50).

Although all cDCs share the same precursor cells and many characteristics, they can be divided in two types: cDC1 and cDC2.

#### Conventional DC1 (cDC1)

Peripheral blood myeloid cDC1 are a rare population of DCs [0.05% of PBMCs (51) or one tenth the amount of cDC2 (52, 53)]. They are identified by the expression of high levels of CD141 (BDCA-3) and XCR1 (54), together with expression of C-type lectin-like receptor (Clec)9A and cell adhesion molecule 1 (CADM1) (28, 29). cDC1 express CD13 and CD33 in common with cDC2, but lower CD11c, CD11b, and SIRPa (CD172). They detect intracellular dsRNA and DNA thanks to their high expression levels of TLR3, TLR9, and TLR10 (28, 55, 56), leading to IRF3-dependent production of type I IFNs and IL-12 (57, 58). Secretion of IL-12 promotes Th1 and NK cell responses. However, cDC1 are most characterized by their superior ability to cross-present and efficiently prime CD8^+^ T cells via MHC class I (51, 59). Interestingly, it has been shown that cDC1 are constitutively resistant to productive viral infection (60).

#### Conventional DC2 (cDC2)

cDC2 cells are the major subset of myeloid DCs in blood, and are characterized for being potent stimulators of naïve T cells. They are identified by the expression of specific markers such as CD1c, CD2, FceR1, SIRPa, and myeloid antigens CD11b, CD11c, CD13, and CD33. Also, transcriptional profiling studies have recently identified CLEC10A (CD301a), VEGFA, and FCGR2A (CD32A) as cDC2 markers (60). cDC2 resemble monocytes in the expression of a wide range of lectins, TLRs, and NOD-like receptors (28). The lectins expressed by cDC2 include Clec4A, Clec10A, Clec12A, and DEC205 (Clec13B) (61, 62). cDC2 express TLR2, 4–6, 8, and 9 and, upon stimulation, they produce several soluble factors such as TNF-a, IL-1, IL-6, IL-8, IL-10, IL-12, IL-18, and IL-23 (63, 64) and chemokines including CCL3, CCL4, and CXCL8 (28, 65). Due to the wide range of factors produced and strong cross-presenting abilities, cDC2 promote a potent activation of Th1, Th2, Th17, and CD8^+^ T cell responses (66).

Interestingly, a population of CD11c^+^ DCs has been found in the anogenital tissue. These cells are transcriptionally very similar to cDC2, but present a higher expression of CD4 and CCR5, which could explain the higher susceptibility to HIV-1 productive infection and consequent transmission to T cells (67). Their presence in the anogenital tissues (where HIV-1 infection occurs) may be key in the initial spread of the virus and establishment of the infection during sexual transmission.

DCs can be obtained *in vitro* from CD14^+^ monocytes after stimulating with granulocyte/macrophage colony-stimulating factor (GM-CSF) and IL-4 (68). These cells express the same maturation and function markers (69), and resemble the behavior of cDC2.

## Role of Dendritic Cells in HIV-1 Infection

As it was mentioned before, DCs are the APCs par excellence. Their most recognized role is to process Ags and present them on MHC on the cell surface, together with co-stimulatory molecules, aiming to present them to T cells and activate a specific and efficient immune response. In order to achieve their objective, once they find an Ag and become mature, they travel to secondary lymphoid tissues, where most resting or naïve T cells are accumulated.

The APC role of DCs that have been in contact with HIV-1 is a two-faced job. On one side, DCs can activate an immune response against HIV-1 in very early stages of infection, by presenting HIV-1 peptides to lymphocytes and thus promoting differentiation, activation and proliferation of HIV-1 recognizing T and B cells. However, HIV-1 also uses DCs to travel to T cell clusters and boost infection of T cells, through a process known as trans-infection which was first described in 1992 (70).

**Figure 1:**
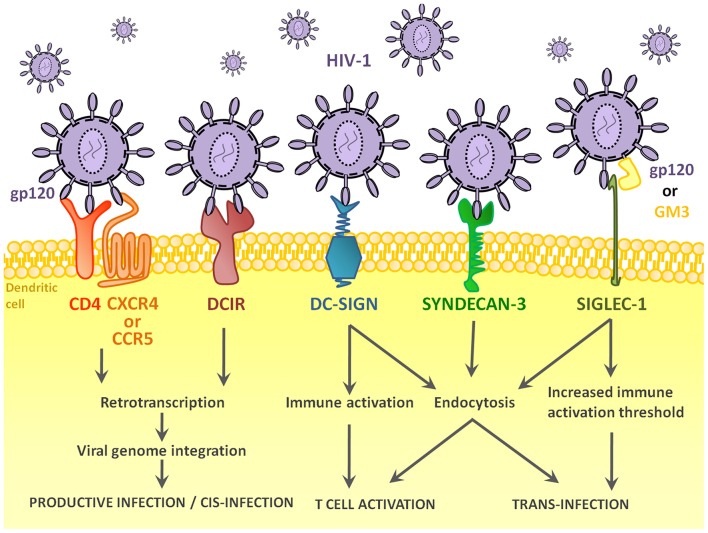
Receptors and pathways implicated in the entry of HIV-1 into DCs. HIV-1 binds several different DCs surface receptors, which determines the fate of the virus. Binding to conventional HIV-1 receptor CD4, or DC-specific DCIR leads to productive infection of the cell in a very small percentage of DCs. In most cases, HIV-1 enters the DC via endocytosis after binding DC-SIGN or other receptors, namely, Syndecan-3 or Siglec-1. Binding to these receptors can lead to trans-infection or immune recognition and consequent T cell activation.

**Figure 2:**
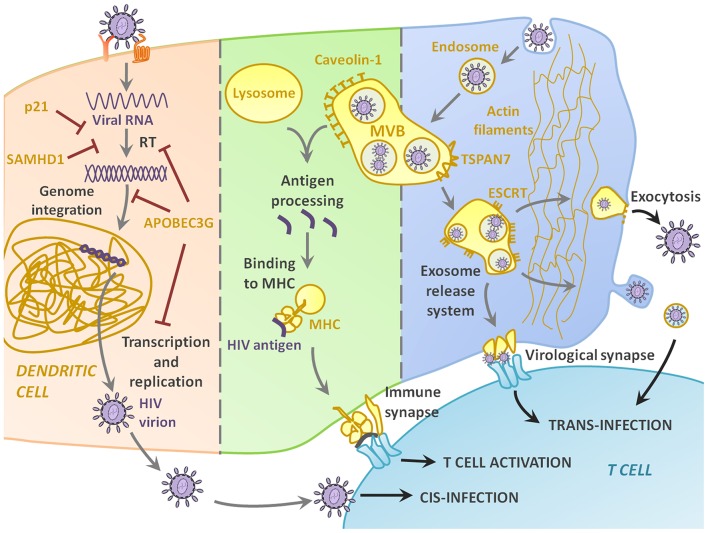
Intracellular pathways and molecular partakers of HIV-1 trip inside DC. Only a small percentage of HIV-DC interactions lead to productive infection, thanks to intracellular molecular defense at different stages of the infection, including and highlighting the antiviral effect of p21, SAMHD1, and APOBEC3G. Most of the times, however, the virus enters the DC by endocytosis and accumulates in multivesicular bodies (MVB). If the MVB fuses with the lysosome, the virus is recognized as antigenic and processed, resulting in viral peptides binding to MHC and showing in the membrane for AG presentation to T cells. The key and most interesting role of DCs in HIV-1 infection is their function as Trojan Horse, as the virus in the endosome can use the cells as a mean of transportation to the lymph nodes, and then be released either through the virological synapse, or via exocytosis.

**Figure 3:**
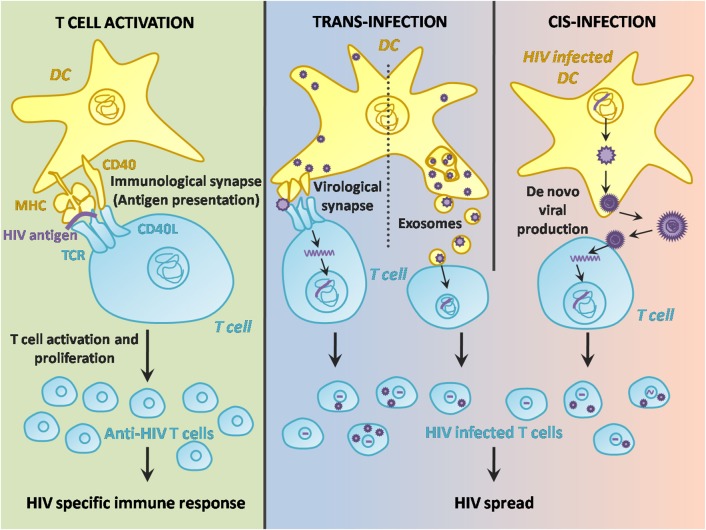
Two-faced role of DCs in HIV-1 infection after T cell contact. DC contact with T cells through the immunological synapse results in Ag presentation and a specific HIV-1 immune response. However, the DC-T cell interaction may also facilitate the transmission of the virus either from an infected DC cell to the surrounding T cells in the lymph nodes, or from a “carrier” DC via exosome release or infectious synapse.

The pathway leading to DC-T cell contact can be divided in three steps: HIV-1 capture by DCs (Figure 1), intracellular processing and trafficking of captured virus (Figure 2), and connection with T cells (Figure 3).

### HIV-1 Capture by Dendritic Cells

The recognition and capture of HIV-1 by DCs is a key step to determine the interaction between virus and immune system. The cell receptor that first recognizes HIV-1 and captures virus will determine the fate of cell and virus (71) (Figure 1). The main players are CD4 and DC-SIGN, leading the first one to conventional infection and the latter to either trans-infection or Ag presentation.

CD4 is the HIV-1 receptor par excellence. Binding of HIV-1 to this receptor, and a CCR5 or CXCR4 co-receptor will cause the virus to enter the cell and follow its conventional viral cycle, including retrotranscription of its genetic material, integration in the host genome and eventually, viral production. Productive infection of DCs is a rather rare event, 10- to 100-fold less frequent *in vivo* than CD4^+^ T cell HIV-1 infection (72). In fact, an *ex-vivo* study using DCs from healthy donors found that on average only 1–3% of both cDCs and pDCs are susceptible to HIV-1 infection, as observed by intracellular staining of HIV-1 protein p24 (10). However, even DC infection levels of <1% are more than sufficient to cause an explosive viral infection in CD4 lymphocytes (73).

An alternative DCs-specific HIV-1 binding protein was first described in 2000 (74). The C-type lectin receptor (CLR) DC-SIGN, which is highly expressed on cDCs located in mucosal tissues, recognizes mannose and fucose on a wide range of pathogens, including the high-mannose oligosaccharides of HIV-1 gp120. DC-SIGN is the key to understanding the dual and ambiguous role of DCs in HIV-1 infection. It acts both as HIV-1 receptor, enhancing trans-infection, and as pattern recognition receptor (PRR), enabling the development of a specific adaptive immune response (75). The fate between these two possibilities has been shown to depend on the N-glycan composition of HIV-1 (76). This was first noted after comparing the fate of normally glycosylated HIV-1 (containing a heterogeneous glycan composition) with that of oligomannose-enriched virus, and observing that the modified virus leads to enhanced viral capture by DCs, enhanced viral degradation and more efficient presentation to CD4^+^ T cells, instead of leading to HIV-1 transmission. Different viral strains differ in their glycan composition, and more recent studies have proven its relevance, by showing that viral strains containing high-mannose glycans were more efficiently bound to DC-SIGN, degraded and presented as Ags (77, 78). Interestingly, the glycan composition of the HIV-1 envelope, and consequent sensitivity to lectins, also correlates with their sensitivity to neutralizing antibodies (Abs) (78, 79).

Binding of HIV-1 to DC-SIGN has been linked to trans-infection rather than to productive HIV-1 infection of DCs. The main mechanism for trans-infection is the transmission of viral particles through the infectious synapse between cDCs and T cells, which will be explained in the next sections. Leukemia-associated Rho guanine nucleotide exchange factor (LARG) is essential for the formation of synapse and has been shown to be activated by DC-SIGN triggered by HIV-1. LARG activation then leads to recruitment and activation of the small GTPase Rho A, but the consequences of this signaling have not been fully understood yet (80). Accordingly, a recent study (81) showed that blockage of DC-SIGN by binding to other proteins such as surfactant protein D, which is secreted by epithelial cells to mucosal surfaces, suppresses binding to gp120 and thus decreases the transmission to lymphocytes.

On the other hand, DC-SIGN bound HIV-1 can be internalized and processed for Ag presentation both to CD4^+^ T cells via MHC-II (82) and to cytotoxic T cells via MHC-I (83). The signaling cascades initiated after HIV-1 binding to DC-SIGN lead to an increase in IL-10 production, which, due to its role in differentiation of naïve T cells into helper cells, is in concordance with the APC role of cDCs. DC-SIGN triggering activates RAF1, a serine/threonine protein kinase, which induces the phosphorylation of NF-κB subunit p65 at Ser276. This phosphorylation enables the binding of histone acetyl transferases CREB-binding protein (CBP) and p300 to p65 and, thus, its acetylation, which results in an increased activity of the NF-κB transcription factor, this way increasing the transcription and expression of IL-8 and IL-10 (84). Surprisingly, phosphorylated p65 recruits pTEF-b, containing cyclin T1 and CDK9, to HIV-1 long terminal repeats, promoting transcriptional elongation, and thus, favoring productive replication if the viral genome has already been integrated in the DC genome (85). Even though TLR signaling is not required for RAF1 activation by DC-SIGN, NF-κB translocates to the nucleus as a consequence of TLR-4 and TLR-3 signaling pathways, suggesting a crosstalk between DC-SIGN and TLR signaling (84). The combined effects of Ag sensing by TLR-3, TLR-4, NOD2, and DC-SIGN have been dissected in a recent study (86) strongly proving that the ligand-driven triggering of these PRRs reduces viral replication and increases the ability of cDCs to stimulate HIV-specific cytotoxic T cells. Although, RAF1 is a well-known component of the RAF1-MEK-ERK signaling cascade (87) and HIV-1 has been linked to ERK activation (88), binding of mannose-containing pathogens by DC-SIGN does not trigger ERK activation, and to the extent of our knowledge, the involvement of DC-SIGN in ERK activation has not been proven, so it is unlikely that DC-SIGN is the initiator of the cascade leading to ERK activation.

Additionally, other receptors could be implicated in the binding of HIV-1 to DCs. For example, cell-surface heparan sulfate proteoglycan, syndecan-3, has been shown to bind to HIV-1 gp120 protein and to be involved in the capture of the virus (89). Another recently discovered C-type lectin receptor, named DCIR (for DCs immunoreceptor) has been demonstrated to bind to HIV-1, to participate in its capture and to be involved in processes leading to the productive infection of monocyte-derived DCs, and thus, to cis-infection of T cells (90). Finally, the sialic acid-binding immunoglobulin-like lectin Siglec-1 was shown to bind the host-derived ganglioside GM3 that is incorporated into the viral membrane and is a key interaction involved in HIV-1 transfer to CD4^+^ T cells via trans-infection (91–93). Siglecs are immune modulatory transmembrane proteins that bind sialylated carbohydrates and set the activation thresholds for immune response. HIV-1, similar to many other pathogens, has evolved to incorporate sialic residues on gp120, and can not only bind to Siglec-1 on DCs and monocytes/macrophages through gangliosides on the viral lipid envelope, but also to Siglec-7 on NK cells and monocytes/macrophages (94), as has been reviewed by Mikulak et al. (95). The binding of HIV-1 to Siglec-1 also increases the entry to DCs, contributing to HIV-1 dissemination.

The entry way of HIV-1 into cells has been historically believed to be via membrane fusion after receptor and co-receptor binding. However, recent studies have proven that HIV-1 can enter the cell via dynamin-dependent endocytosis, and that membrane fusion can occur inside the cell, starting controversy (96). These different entry pathways could depend on cell type, and in the case of DCs, there is consensus in that the virus can either fuse at plasma membrane or be endocytosed, leading to productive infection or internalization and trans-infection of T cells, respectively (97). The endocytosed virus has been demonstrated to cause productive infection of CD4^+^ T cells (98). HIV-1 entry to DC by endocytosis is also the path leading to lysosomal fusion, processing and Ag presentation.

Pritschet et al. (99) highlighted the importance of the endocytic pathway in pDCs when they demonstrated that endocytosis, but not membrane fusion, increased the IFN-α production. They treated pDCs with dynasore, a GTPase inhibitor that rapidly inhibits dynamin and thus, endocytosis, or with fusion inhibitor T-20, and observed that only the former decreased IFN-α production.

Interestingly, binding of HIV-1 particle to C-type lectin receptors, mainly the aforementioned DC-SIGN, but also the Langerhans cell specific langerin, preferentially route the HIV-1 toward the endocytic pathway. Langerhans cells are DCs that reside in the epithelium characterized by their expression of langerin and the presence of endosome-related Birbeck granules in their cytoplasm. Binding of HIV-1 to langerin leads to internalization via caveolin-1 positive vesicles, linked to lysosomal degradation pathway, thus representing a restriction mechanism for HIV-1 infection (100).

The most recent studies in DCs, suggest that HIV-1 fusion occurs mostly at cell surface, instead of endosomic vesicles, and depends on the amount of CD4 expressed (101). Low levels of CD4 expression limit the fusion and divert DCs-HIV-1 interaction toward the competing binding to DC-SIGN and the consequent not productive pathway. The interaction of DCs with HIV-1 is strongly determined by the maturation state of the cells. Viral fusion is even lower and shows slower kinetics in mDCs when comparing with iDCs (102).

### Intracellular Trip

#### Dendritic Cell Infection or Lack Thereof

As it has been mentioned before, the interaction between HIV-1 and host cell can lead to productive infection, but more often than not, HIV-1 follows other alternative pathways. Some intracellular factors that play a key role in determining the fate of such interaction have been discovered (Figure 2). Intracellular antiviral responses represent a first line of natural defense in preventing infection as proved by the fact that HIV-1 has developed efficient counteracting measures.

Similar to viral fusion, viral replication is less effective in DCs than in CD4^+^ T cells, but can still occur in DCs. iDCs can be productively infected, although only by R5 virus, while mDC can internalize both R5 and X4 HIV-1 strains, but the replication is blocked (103). It is controversial whether the transcription in mDC is blocked at the step of reverse transcription (104) or inhibited post-integration (105). A study from 1999 showed that the transcription blockage in mDC is surpassed after binding to T cells, via binding to CD40L, allowing and promoting cis-infection (106).

Despite binding and internalization by DCs, HIV-1 fails to induce a strong innate immune response or inflammation. The mechanisms that HIV-1 uses to evade detection inside the DCs are still to be fully understood. The inflammatory signaling pathways in DCs are stimulated by HIV-1 after binding to TLR-8. Using a phosphoproteomics approach followed by a siRNA screen, a recent study found that HIV-1 exploits the dynein motor protein Snapin, a natural cellular inhibitor of TLR-8 signaling and a general regulator of endosome maturation, by enhancing dynein expression. The increase in Snapin expression facilitates the accumulation of HIV-1 particles in endosomes containing low TLR-8, hence evading the sensing and inflammatory response (107).

The low HIV-1 infection is presumably due to low expression of CD4 receptor and co-receptors on the surface of the DCs, fast and efficient degradation of internalized virus, limited availability of dNTPs on non-cycling cells, and the expression of host factors that could block HIV-1 infection and/or replication (108).

The fact that HIV-1 replicates inefficiently in non-dividing cells, and the discovery that the infection can be enhanced by Vpx [a protein encoded by HIV-2 and several lineages of simian immunodeficiency virus (SIV)], pointed at the existence of a cellular HIV-1 restriction factor (109). Sterile alpha motif and HD-domain containing protein 1 (SAMHD1) was first described in 2011 as the restriction factor that gives non-dividing myeloid cells certain resistance against HIV-1 infection (110, 111). It was identified by mass spectrometry studies of proteins immunoprecipitated from cells expressing or not Vpx. Vpx was later shown to interact with the C-terminal domain of SAMHD1, driving it to E3 ubiquitin ligase complex, leading to proteosomal degradation (112). SAMHD1 is expressed in the majority of the nucleated hematopoietic cells, and it is especially abundant in the HIV-1 target cells residing in the anogenital mucosa (113). Keeping in mind that HIV-1 does not contain Vpx, all this information brings up the questions of how HIV-1 avoids the SAMHD1 barrier, what is the role of SAMHD1 in HIV-1 infection, and whether it could contribute to the inefficient immune response against HIV-1 (114).

SAMHD1 is a phosphohydrolase (115) that dephosphorylates, and thus depletes, the pool of deoxynucleotide triphosphates (dNTP) (115, 116) to a level below that required for HIV-1 reverse transcription (117). Structural studies have shown that SAMHD1 works as a highly efficient catalytic tetramer, which forms after binding to GTP and dGTP (118–121). This allosteric regulation by GTP warrants a close communication and coordinated regulation with the ribonuclease reductase (the enzyme responsible for *de novo* dNTP synthesis) in order to maintain balanced intracellular dNTP levels. As can be intuited, SAMHD1 regulation is cell-cycle dependent, and it has several means of control. Mainly, it is inhibited by phosphorylation in Threonine 592 by the complex of cyclin A with cyclin-dependent kinase 1 (CDK1) and CDK2 (122–124). Binding of single-stranded nucleic acids to dimerizing interface inhibit formation of the catalytic tetramer (125), and also oxidation caused by proliferation-induced hydrogen peroxide forms disulfide bonds and blocks catalytic activity (126).

These regulatory mechanisms keep SAMHD1 inactive during the G1 and S phase of the cell cycle in proliferating cells and explain why HIV-1 infects dividing cells more efficiently. The same study that showed that SAMHD1 is expressed in most tissues and hematopoietic cells, also pointed out that, predictably, activated CD4^+^ T cells contain the phosphorylated form pSAMHD1 (T592), whereas cells, which are not HIV-susceptible such as resting T cells and macrophages, carried the HIV-restrictive unphosphorylated protein (113). In macrophages, cyclin L2 has been found to bind to SAMHD1, leading to its degradation and thus controlling its abundance (127). Knockdown of cyclin L2 highly decreased HIV-1 replication in macrophages, but not in cyclin cells, which suggest the possibility that it might also be found in DCs.

Several functions and roles related to immune response have been attributed to SAMHD1, including down-regulating type I interferon response, which depends on the residue G209 of SAMHD1 (128), and blocking the replication of virus that uses retrotranscription in their replication cycle (retroviruses and hepatitis B virus) (129, 130). Although SAMHD1 had been reported as a type I interferon (IFN)-inducible protein, its expression or phosphorylation levels have been demonstrated to show no variation after IFN stimulation (131). However, its relationship with IFN is still controversial. Other studies claim that the constitutive signaling through the type-I IFN receptor in pDC blocks the degradation of SAMHD1 and counteracts the effect of Vpx (132).

SAMHD1 has a dual enzymatic function, degrading not only dNTPs (dNTPase activity) but also viral nucleic acids (RNase activity). The nuclease activity is also implicated in HIV-1 restriction (133), although there is no consensus about whether it is required (134) or not (135) for HIV-1 restriction. Recent studies on this topic showed that an increased expression of cyclin-dependent kinase inhibitor p21^Waf1/Cip1^ (p21) also decreases the size of the intracellular dNTP pool by suppressing several enzymes involved in dNTP synthesis. Interestingly, p21 blocks SAMHD1 phosphorylation and promotes its dNTPase-independent antiviral activity without affecting its dNTPase activity (136). The expression of p21 is low in responding iDC, and increases gradually during differentiation (137). This correlates with the fact that some iDCs can be productively infected, while the viral replication is blocked in the mDCs that internalized the virus (103). Altogether, induction of p21 may result in an effective inhibition of HIV-1 replication in DCs, appearing to be a key regulator of HIV-1 infection and a potential target for drug design.

Another innate antiviral factor was first isolated in 2002, and identified as APOBEC3G (apolipoprotein B mRNA editing enzyme catalytic polypeptide-like 3G) (138). It was then described to act at the late stages of viral production and to be counteracted by the HIV-1 protein Vif (139). Two other components of APOBEC family, APOBEC3F, and APOBEC3B, exhibit anti-HIV-1 activities (140). Interestingly, APOBEC3G, together with APOBEC3F, can be encapsulated into HIV-1 virions, in absence of Vif, decreasing the infectivity of the virion (141), and it has been long studied as a candidate target for drug discovery (142). Both APOBEC3G and APOBEC3F are expressed in cell populations susceptible to HIV-1 infection, whereas APOBEC3B is not normally expressed in lymphoid cells and has been shown to be resistant to HIV-1 Vif. Therefore, activation of endogenous APOBEC3B gene in primary human lymphoid cells has been proposed as a novel and effective strategy for inhibition of HIV-1 replication *in vivo* (143).

APOBEC3G is a cytidinedeaminase of ssDNA, which means that it binds to ssDNA and catalyzes the deamination of cytidine to uridine, and it exerts its antiviral activity by interfering with the proper replication of viruses. However, in the case of HIV-1, it has been shown that APOBEC3G inhibits infection in two previous steps of the viral cycle. First, it interferes with reverse transcription, in a deaminase independent manner, by blocking tRNA${}_{3}^{\text{Lys}}$ priming, thereby inhibiting the production of early minus-sense ssDNA (144). This implies that it cannot just bind to DNA, but also to RNA, through at least two different described RNA binding sites (145). Nonetheless, five to seven-fold reduction in viral DNA synthesis in the presence of APOBEC3G and absence of Vif only partially accounts for the 35-fold decrease in HIV-1 infectivity, which led to the discovery that there is an additional role of APOBEC3G that results in a five-fold decrease in the amount of integrated DNA. APOBEC3G modifies the processing and removal of tRNA by reducing its efficiency and specificity, resulting in aberrant viral DNA ends, inefficient for integration (146). Vif triggers the poly-ubiquitination of APOBEC3G and targets it for proteasomal degradation (147).

Despite being present in most cell types, APOBEC3G has an especially important role in DCs. Pion et al. (148) proved in 2006 that APOBEC3G levels correlate with HIV-1 infection in DCs. They showed that the small subset of iDC susceptible to HIV-1 infection were deficient in APOBEC3G, and they also noted that APOBEC3G levels increased during maturation, which further reduces susceptibility to infection. Likewise, APOBEC3G expression is up-regulated by DCs stimulation with CD40 and CCR5 (149), and poly (I:C) and TNF-α (150), which are known to induce DCs maturation.

Due to the importance of IFN-α in the innate immune response against viral infections, including HIV-1, its effect on APOBEC3G expression appears particularly interesting. A study published in 2013, found that low amounts of IFN-α significantly induced APOBEC3A, F and G expression in monocyte-derived iDC, without causing maturation (151). In the same study, they showed a significantly reduced transmission of HIV-1 from DCs to autologous T cells in the presence of IFN-α, presumably caused by the increased APOBEC expression levels. Similar results were found in pDCs, in which IFN-α increases APOBEC3G, thus decreasing HIV-1 infectivity (152).

APOBEC3G is only enzymatically active when in low molecular mass (LMM) configuration, but its recruitment into high molecular mass (HMM) RNA-protein complexes results in a block in its deaminase function. Activation of CD4^+^ T cells enhances APOBEC3G expression and recruitment into HMM, rendering the cells permissive to HIV-1 infection. On the contrary, maturation of DCs leads to an increased expression and accumulation of LMM APOBEC3G, which then contributes to halting HIV-1 infection (150).

Another characteristic of APOBEC3G which differs in DCs with regards to other cell types is the subcellular location. In CD4^+^ T cells, the expression APOBEC3G increases with activation, but it translocated to the nucleus, which is compatible with the increase in HIV-1 infection susceptibility. In DCs, APOBEC3G is localized in the cytoplasm, where it performs its functions, regardless of the maturation state of cells (153).

Although less efficient than SAMHD1 or APOBEC3G, TRIM5α is another known HIV-1 restriction factor that was first described as a rhesus macaque protein responsible for blocking infection by HIV-1 (154). TRIM5α restricts HIV-1 infection by disrupting the viral capsid and inducing its proteasome-dependent degradation (155–157). Its function in DCs is regulated by SUMOylation and nuclear sequestration (158). Recent studies showed that IFN-α-mediated stimulation of immunoproteasome facilitates the degradation of the viral capsid and inhibition of HIV-1 DNA synthesis by TRIM5α (159), thus suggesting a possible mechanism of action of this restriction factor. However, TRIM5α does not have a strong role in DCs, as it has been shown to only restrict HIV-1 infection upon C-type-lectin-receptor-dependent uptake of HIV-1 (160). DC-SIGN dependent entry of the virus leads to dissociation of TRIM5α from DC-SIGN, and consequent abrogation of restriction.

Interestingly, the effect of HIV-1 productive or restrictive infection on gene expression pattern depends on the maturation state of DCs. A study based on whole-genome microarrays recently found that expression of interferon-stimulated genes involved in control of viral replication, was induced after productive HIV-1 infection of iDCs with Vpx-loaded HIV-1 particles, thus inducing an antiviral state in surrounding cells. On the contrary, in the case of mDCs, productive HIV-1 infection decreased expression of interferon-stimulated genes CXCR3-binding chemokines, suggesting a diminished trans-infection of CD4 lymphocytes. Paradoxically, restrictive HIV-1 infection had the opposite effect on mDCs, increasing the aforementioned gene expression, which would result in lymphocyte attraction and enhanced trans-infection (161).

Summing up, the currently known restriction factors and their mechanisms of action may represent only the tip of the iceberg of mechanisms evolved to protect eukaryotic cells from HIV-1 infections, and to preserve the integrity of host cells and of its genome. Equivalently, SAMHD1, APOBEC3G and TRIM5α are well-known cellular factors that restrain HIV-1 from productively infecting and replicating in DCs, but further research could shed light on other mechanisms or ways to take advantage of these ones with a therapeutic or prophylactic aim.

#### Dendritic Cells as HIV-1 Carriers

Although DC infection is rare, HIV-1 frequently contacts DCs and enters to the cell, harnessing cell migration to reach more infection-prone cells. This pathway is the most favored after HIV-1 binding to DC-SIGN or other C-type lectin receptors, or when the virus enters via endocytosis. In this case, the virus uses the DC as a mere mean of transportation. The endosomes develop into multivesicular bodies (MVBs), and can then follow two paths: lysosomal fusion and degradation, probably leading to antigen presentation, or re-fusion with plasma membrane and release of exosomes or viral particles, spreading the infection.

In the specific case of langerin mediated HIV-1 capture, the viral particles have been shown to accumulate in vesicles in co-localization with langerin and caveolin-1, which was related to the lysosomal degradation pathway (100). A similar pathway was described in pDCs, after dynamin-dependent endosomal entry of HIV-1. The viral particles were found to localize in vesicles containing caveolin; early endosomal Ag 1; RabGTPases 5, 7, and 9, and lysosomal-associated membrane protein 1 (99). These data suggest that HIV-1 containing endosomes also follow the lysosomal pathway, thus triggering Ag presentation and an adaptive immune response. Lysosome fusion and Ag degradation is a key part of antigenic peptide binding to MHC-I or MHC-II and consequent Ag cross-presentation to CD8^+^ or CD4^+^ T cells, respectively (162). Further research on this topic is needed, as promotion of this pathway offers a potential road to anti-HIV drug design.

Binding of HIV-1 peptides on MHC-I molecules is remarkably interesting for the design of a therapeutic vaccine (163). The cytotoxic responses triggered by MHC-I presentation to CD8^+^ T cells have a key role in the specific immune response against HIV-1, and cDCs have been shown to process antigen, present it on MHC-I and generate a specific anti-HIV-1 cytotoxic immune response (164). Intracellular processing is required for optimal presentation, and the ability of DCs to process and present antigen on MHC-I remains intact in DCs derived from HIV-1 infected patients on cART (165). This processing pathway could be exploited for the design of DC-based therapies against HIV-1.

However, HIV-1 avoids lysosomal degradation and exploits the endosomal pathway in DCs, turning it into an efficient and dangerous way to spread to lymph nodes, where it transfers to T cells, its favored target. HIV-1 transfer from DCs to T cells is regulated by actin dynamics (166). Recent studies have revealed the role of TSPAN7, a member of the tetraspanin family which promotes actin nucleation and stabilization via the ARP2/3 complex, in halting HIV-1 internalization in the endosome, keeping it close to plasma membrane, in actin-rich dendrites, favoring an efficient transfer to T cells (167).

TSPAN7 increases the list of tetraspanins related with the HIV-1 infection cycle (168). The HIV-1 budding sites, virological synapses, and virions have long been known to be enriched in the tetraspanin family members CD9, CD63, and CD81 (169). HIV-1 uses these host molecules for its own benefit. CD9 regulates trafficking of MHC-II and its surface expression levels, thus affecting Ag presentation (170). CD81 is associated with SAMHD1, decreasing its activity, increasing the availability of dNTP, and thus enhancing HIV-1 infection (171). CD63 has a dual activity and its function is still not clear. It regulates HIV-1 replication (172) and it is required for reverse transcription (173).

Finally, there is a group of proteins that is booming in the last years, but whose role in the context of DCs and HIV-1 has been overlooked for now. The endosomal sorting complexes required for transport (ESCRT) machinery consists on a group of cytosolic protein complexes that enable membrane remodeling and budding away from the cytoplasm, and thus orchestrate cellular processes such as MVB and exosome biogenesis (174), cytokinesis and viral budding (175). HIV-1 is not an exception, and takes advantage of this tool to be released from infected cells (176, 177). Briefly, HIV-1 gag has been shown to engage tsg101 (178, 179) or ALIX (180) in order to recruit the rest of the ESCRT machinery to the assembly sites where they mediate budding. High-throughput screening is a useful tool for finding inhibitors for these interactions (181). When looking at DCs, the ESCRT machinery has an extra function, as it regulates Ag presentation by MHC class-II (182). The ESCRT proteins drive MHC class-II complex to either lysosomal degradation in non-activated DCs, or transfer to cell surface for Ag presentation in activated DCs, thus standing out as key figures for DC function. It would be interesting to study the specific role of the ESCRT machinery in DCs in presence of HIV-1, as a potential start point for the design of anti-HIV-1 drugs or therapeutic vaccines.

Although the main questions that come to mind when in the rational search of an HIV-1 therapeutic or prophylactic are usually focused on virus-cell interactions and viral replication, the multiple and diverse intracellular molecular players that determine the fate of the virus and the outcome of infection also form a rich and vastly unexplored field with a big potential for drug design. Altering the HIV-1 intracellular pathway may serve to modulate the immune response and control the consequences of the infection, pointing out the importance of a detailed study of all the implicated molecules.

### Dendritic Cell-T Cell Contact

The ultimate function of DCs is to present Ag to the T cells, for which they establish contact either in the mucosa or after migration to the lymph nodes. HIV-1 subverts the Ag presentation process to increase transmission and infection of T cells (Figure 3). Although some DCs can retain viruses up to 6 days, most viruses are degraded within 24 h after exposure, which does not match the timeline of T cell infection in co-culture. A 2-phase transmission pathway has been suggested, initiated by iCD and mCD uptake and transmission of HIV-1 to T cells via trans-infection that decays after 24 h, and followed by a long-term second round of cis-infection after 48 h, when the infection has resulted productive in iDCs, and a new generation of virus is transmitted to T cells (183).

#### Dendritic Cell-T Cell Activation

As we have mentioned before, cDCs are mostly known for their role as APCs. Although the molecular mechanisms remain controversial, the accepted dogma is that cDCs capture Ag by endocytosis, degrade it into peptides after fusion of the endosome with the lysosome, and present it to T cells bound to MHC complex. Recognition of MHC bound Ag, together with binding to co-stimulatory molecules on the cDC surface, in a molecular cluster known as the “immunological synapse,” induces specific T cell responses, and thus, the adaptive immune response. The state of knowledge about this process has been reviewed in other recent publications (162, 184–186).

In spite of the extensive research and accumulated knowledge in this field, there are still a lot of questions that remain to be answered and are of great interest for therapy design, among other things. Hence, more detailed molecular pathways and alternative circumstances are being explained. A study from 2018, for example, described a new pathway leading to initiation of adaptive immune responses *in vivo*, which occurs after the infective agent has bypassed capture by the innate immune cells at the infection site (187), providing evidence of the existence of an alternative lymphocyte activating pathway that may function in parallel or as a backup to the conventional pathway. They found that the Ag can travel to lymph nodes, where it is phagocytosed by macrophages, causing their death, and the debris released activates Ag presentation by monocytes in the blood. Further studies about the factors contained in the debris that enhance Ag presentation could provide clues in the search of an HIV-1 therapeutic vaccine.

In the case of HIV-1, cDCs from infected patients are unable to generate lymphocyte activation and proliferation *in vitro*, even when they show all the signs of activation (42). However, cDCs from elite controllers of HIV-1 infection have been found to effectively prime T cell responses (188). These recent results corroborate their previous findings that DCs from elite controllers produced type I IFN in a rapid and sustained way, expressed less SAMHD1, and accumulated viral reverse transcripts after exposure to HIV-1. Those signs of improved cell-intrinsic immune recognition of HIV-1 translated into a strong stimulation of a specific CD8^+^ T cell response, suggesting that improved DC-T cell Ag presentation contributes to control of HIV-1 infection in elite controllers (189).

Recent studies are shedding some light on the mechanisms of induction of HIV-1 specific T cell responses by DCs. The programmed cell death ligand 1 (PD-L1) is highly expressed in highly stimulatory cDCs, and it has been shown to have an enhancing role in priming naïve T cells and activation of CD8^+^ T cells into effector T cells, while it has a negative role in later phases, as it decreases the magnitude of memory HIV-specific cytotoxic T cell responses (190). These findings contradict the previous suggestion that PD-L1 expressing DCs in the lymph nodes may hinder the generation of HIV-specific T cell response (191).

Manipulating cross-presentation is a promising tool for effective vaccine design against cancer and infectious diseases, including HIV-1. For this reason, further exploring and describing the mechanism and main molecular partakers of DC-T cell crosstalk is indispensable in the search of DC based vaccines.

#### Dendritic Cell-T Cell HIV-1 Transmission

##### Trans-infection via infectious synapse

The first studies about DC-T cell HIV-1 transmission claimed that cell-to-cell contact was necessary for the T cell infection to occur (192). This fact was corroborated by several later studies, and the contact phase was named “infectious” or “virological” synapse (193). Although the exact molecule composition and structure of the infectious synapse has not been fully described yet, it is believed to be similar to that of the immunological synapse, through which the APCs present Ag and activate T cells (194, 195). Interestingly, DC-SIGN is detected in and essential for the formation of the infectious synapse, as demonstrated by Arrighi et al. (196, 197) in several experiments suppressing the expression of DC-SIGN and showing the lack of synapses and inefficient HIV-1 transmission. However, it has been reported that in this DC-SIGN mediated HIV-1 transmission, the expression of MHC class II molecules on virus donor cells is not required, setting considerable differences with the immunological synapses (198).

Besides being essential for the formation of the synapse, DC-SIGN binding to HIV-1 particle in the first place also sets a signaling cascade that favors the trans-infection pathway over productive infection or viral degradation. DC-SIGN is known to associate with LARG, whose activation promotes activation of RhoA and the focal adhesion molecules FAK, Pyk2, and paxillin, all of them structural components of the infectious synapse (199).

After binding to DC-SIGN, among other receptors, HIV-1 enters the cell via endocytosis, and remains in MVBs inside the cell. Contact of cDCs with CD4^+^ T cells causes the internalized vesicles containing the HIV-1 particles to migrate to cell-cell junction, facilitating the transfer of virions across the synapse (193). This appears to be more efficient with mature than immature monocyte-derived DCs (183).

Studies comparing HIV-1 non-progressors with HIV-1-infected progressors and HIV-1-seronegative donors showed that cDCs from HIV-1 non-progressors failed to mediate trans-infection, highlighting the importance of this process for HIV-1 infection and dissemination. They also linked the lack of trans-infection to lower cholesterol levels and increased expression of the reverse cholesterol transporter ABCA1 (ATP-binding cassette transporter A1) in the DCs, but not in T cells, thus opening the door to new therapeutic approaches involving lipid metabolism enhancement (200).

Trans-infection via the virological synapse is more efficient for R5-tropic HIV-1 than X4-tropic HIV-1 strains (201). Independently of the viral entry to DCs or productivity of the infection, co-culture of CD4^+^ T cells with R5-infected cDCs resulted in a higher viral expansion than co-culture with X4-infected cDCs. These results were also found to depend on T cell activation state. The maturation state of DCs also dictates the efficiency and selectivity of the viral transmission. mDCs are more efficient than iDCs in transferring HIV-1 to T cells, partially due to the concentration of viral particles at the virological synapse that is more frequently observed in mDCs (202). Studies on DCs activated by different maturation factors showed that iDCs and CD40L-induced mDCs were more susceptible to productive infection and lead to cis-infection, while LPS and TNF-α-induced mDCs mediated efficient trans-infection of CD4^+^ T cells (203). One very important reason to consider halting trans-infection in the search of new effective anti-HIV-1 therapies is the fact that this mechanism of T cell infection is insensitive to the antiretroviral drugs (204). So even under cART or pre-exposure prophylaxis (PreP), HIV-1 could spread to T cells and increase the reservoir, thus hindering the so desired cure.

##### Trans-infection via infectious exosomes

Wiley and Gummuluru discovered an HIV-1 trans-infection pathway mediated not by cell-to-cell contact but by DC-derived exosomes (205). They reported that HIV-1 particles that were captured by iDCs and rapidly endocytosed to tetraspanin-rich compartments could be constitutively released to extracellular milieu in association with exosomes (HLA-DR1^+^, CD1b^+^, CD9^+^, and CD63^+^ vesicles). They also discovered that the HIV-1 particles associated with exosomes from DCs could fuse with target-cell membranes, and were 10-fold more infectious than cell-free virus particles.

Exosomes are small vesicles (50–200 nm) containing genomic, proteomic or lipid cargos, which are released to the extracellular milieu by a broad range of cell types and facilitate cell-cell communication (206). In particular, DC-derived exosomes have been shown to be remarkably efficient at HIV-1 trans-infection. This could be explained by the fact that the exosomes carrying the virus also carry infection enhancing factors, including T cell activators and binding molecules. MHC class II molecules are detected in exosomes, which could be sufficient to activate T cells, increasing their susceptibility to HIV-1 infection (207). A comparative study demonstrated that the presence of fibronectin and galectin-3 in exosomes from DCs was key for HIV-1 trans-infection of T cells, as blockage of both receptors significantly inhibited this process (208).

Recent studies suggest that exosomes from HIV-1 infected DCs can not only activate and infect resting CD4^+^ T lymphocytes via trans-infection, but also reach and reactivate the HIV-1 reservoir, further boosting the progression of infection (209). Those studies further corroborate an almost contemporary publication that describes how exosomes released by uninfected cells already activate transcription of latent reservoirs (210).

Years before the exosome-bound HIV-1 trans-infection pathway was described, exosomes were already in the spotlight of retroviral infection through the widely accepted “Trojan exosome hypothesis” (211). This hypothesis postulates that retroviruses, including HIV-1, exploit the cell-encoded machinery of vesicle traffic and exosome exchange for both the biogenesis of viral particles and mode of infection and spread. Strong evidence supporting this hypothesis is the huge similarity between exosomes and HIV-1 particles regarding their host cell lipid and protein composition and biogenesis. Both particles contain higher levels of cholesterol and glycosphingolipids than the plasma membrane (206, 212). Both exosomes and HIV-1 particles are enriched in many protein components in comparison with the plasma membrane, including tetraspannins (213), GPI proteins and Lamps (214, 215). HIV-1 also exploits the mechanisms that target proteins to MVBs, previous to formation of exosomes. HIV-1 protein Gag not only binds to exosome biogenesis factors, such as cyclophilin and tsg101, but it also forms aggregates. It is N-terminally myristoylated and monoubiquitylated, which are known MVB targeting mechanisms (216). Even more relevant evidence supporting the Trojan exosome hypothesis is the observation of infection independent of *Env* proteins and cell receptors (217, 218). Although depletion of HIV-1 *Env* decreases its infectivity to <1%, *Env* depleted HIV-1 particles still infect CD4^+^ and CD4^−^ cells with the same efficiency. This could be explained by the exchange of exosomes, especially common among immune cells as a communication system for immune surveillance (219, 220), which after being released into the extracellular milieu can fuse with membranes of neighboring cells by a clathrin-mediated mechanism.

In spite of the many similarities regarding biogenesis and molecular composition between HIV particles and exosomes, the presence on exosomes of T cell activators and binding molecules, such as HLA-DR, explains the higher infectivity of HIV carrying exosomes in comparison with free HIV particles (207). As HIV-1 can only infect dividing cells, T cell activators present on exosomes allow the HIV-1 virus to retrotranscribe and infect the T cell after exosome binding. On the other hand, binding molecules increase the affinity of the particle for the host cell, and also allow the HIV-1 virus to enter the T cell, sometimes without even binding to its receptors.

The study of exosomes has increased exponentially recently, and there is only new evidence of the multiple and ambiguous role they play during HIV-1 infection. Some studies, for example, suggest that exosomes carrying CD4 or DC-SIGN receptors compete for binding to the virus, thus pointing to a protective role of exosomes against HIV-1 spreading (221, 222). However, most of the new findings are related to ways by which HIV-1 hijacks the exosomal system and increases infection (223). Surprisingly, HIV-1 binding to DCIR in DCs, which leads to productive infection as explained before, also triggers the release of exosomes containing the pro-apoptotic protein DAP-3, which increased spontaneous apoptosis in uninfected CD4^+^ T cells (224).

Although the relative importance of this pathway remains to be determined, it definitely also contributes to infectious synapse mediated trans-infection by providing a pathway by which the intracellular virus reaches the synapse, avoiding its degradation in the lysosomal pathway.

##### Cis-infection after viral replication in dendritic cells

Trans-infection can persist up to 24 h after HIV-1 binding to the surface of DCs, with a peak around 6 to 12 h, but the majority of the virus is degraded after that time by fusion with the lysosomal compartment in DC. As DCs require around 12–24 h to migrate to the lymph nodes, some scientists claim that it is unlikely that trans-infection is an effective “Trojan horse” for the virus, and that it does not explain the peak of CD-T cell transmission observed after 24 h in co-culture. The second phase of HIV-1 transmission is believed to imply DC productive infection, which, although less efficient than in CD4^+^ T cells, has a key role in long-term HIV-1 transmission (183). *In vitro*, cis-infection is more efficient than trans-infection. This is probably due to a combination of factors including a higher concentration of viral particles in each donor cell, longer duration of HIV-1 transfer (with a peak around 72–96 h), long duration of viral production and cell survival up to 40 days (225), and the need for only less than 0.1% HIV-1 infected DCs.

HIV-1 protein Nef plays an important role at enhancing cis-infection. By inhibiting DC-SIGN endocytosis and thus upregulating surface levels of DC-SIGN in HIV-1-infected DCs, Nef promotes lymphocyte clustering and viral transmission (226). Not only does Nef facilitate DC-T cell contact, but it also promotes T cell activation, which is necessary for HIV-1 infection. A study comparing the effect of WT HIV-1 vs. Nef-mutated HIV-1 on the activation of resting CD4^+^ T cells found that Nef was required for T cell activation and productive HIV-1 infection (227). Contradictorily, Nef expression in HIV-1 infected DCs induces the expression of the interferon-induced protein tetherin (228), which restricts virion release (229–231) and cell-to-cell infection (232).

One of the biggest challenges in studying *de novo* HIV-1 production is the lack of tools to distinguish between cytosolic immature and endocytic mature virus. Turville et al. (233) developed an assay that allows differentiating between the newly synthesized viral particles and the mature virus traveling through the endocytic path. They developed a modified infectious HIV-1 construct that could be stained with biarsenical dyes and allowed the restricted observation of newly synthesized uncleaved gag protein. Combination of this system with HIV-1 gag antibodies, which stain mature virions, can be used to track viruses at different maturation stages.

As it has been mentioned before, mDCs are more permissive of HIV-1 replication than iDCs, and also display an increased surface expression of CXCR4, favoring the infection by X4 tropic viruses. Thus, it seems logical that the maturation stage of DC plays a crucial role in HIV-1 infection, spreading of viral strains and disease progression. This was shown by a study promoting DC maturation, by treatment of DCs with dectin-1/TLR2 and NOD2 ligands, which resulted in an increase of cis-infection of autologous CD4^+^ T cells by X4-tropic HIV-1 (234).

## Implications and Conclusions

The modification of DCs and their use as immunotherapy is increasing in the last years, especially in the field of cancer therapies (235). DC-based therapy has been shown to be safe and feasible in several phase I/II clinical trials (236–238). Due to their many implications and key role in adaptive immune system activation and also in HIV-1 infection, DCs are also promising immunotherapy candidates in the search of an HIV-1 functional cure or vaccine.

Despite the growing knowledge in DC biology and HIV-1 infection, the exact mechanisms that determine the type of response generated are still poorly understood and further research is required. Understanding these immune behaviors would empower rational frameworks for the design of immune modulators working on a systems level that serve a prophylactic or therapeutic purpose.

Future therapies could work at any stage and take advantage of the peculiarities of DCs for a highly specific and targeted treatment. For example, specifically targeting DC-SIGN has been achieved thanks to advances in drug design (239). This could be useful for *in vivo* targeting of drugs to DCs. According to what has been summarized in this review, harnessing certain entrance mechanisms could determine the fate of the HIV-1 or Ag, and could promote Ag presentation instead of viral spread (162). Natural DC responses can be manipulated in order to create a specific T cell response, both cytotoxic and humoral. In fact, DC membrane vesicles are already being used as a delivery platform for CD8^+^ activation (240).

All these steps of DC function should be exploited but the design of controlled, safe and efficient DC-based therapies require more molecular, and systems knowledge. Further studies in the field of DC modulation, Ag presentation and DC-HIV-1 interaction are essential to set the bases for the so desired HIV-1 functional cure.

## Author Contributions

Both authors conceived the review and have participated in writing, graphics, review, and discussion of the article. Both authors read and approved the final version or article.

### Conflict of Interest

The authors declare that the research was conducted in the absence of any commercial or financial relationships that could be construed as a potential conflict of interest.
